# Structure, Synthesis, and Catalytic Performance of Emerging MXene-Based Catalysts

**DOI:** 10.3390/molecules29061286

**Published:** 2024-03-14

**Authors:** Zhengxiang Sun, Rui Wang, Vitaly Edwardovich Matulis, Korchak Vladimir

**Affiliations:** 1 School of Environmental Science and Engineering, Shandong University, Qingdao 266237, China; 2 Scientific-Research Institute for Physical Chemical Problems, The Belarusian State University, 220006 Minsk, Belarus; 3 Semenov Federal Research Center of Chemical Physics, Russian Academy of Sciences, 119991 Moscow, Russia

**Keywords:** MXene, catalytic activity, composite materials, performance comparison

## Abstract

As traditional fossil fuel energy development faces significant challenges, two-dimensional layered materials have become increasingly popular in various fields and have generated widespread research interest. MXene is an exceptional catalytic material that is typically integrated into functional composite materials with other substances to enhance its catalytic-reaction performance. Improving the thermal stability, electrical conductivity, and electrochemical activity, as well as enhancing the specific surface structure, can make the material an excellent catalyst for photoelectrocatalysis and energy-regeneration reactions. The article mainly outlines the structural characteristics, preparation methods, and applications of MXene in the field of catalysis. This text highlights the latest progress and performance comparison of MXene-based catalytic functional materials in various fields such as electrochemical conversion, photocatalysis, renewable energy, energy storage, and carbon capture and conversion. It also proposes future prospects and discusses the current bottlenecks and challenges in the development of MXene-based catalytic materials.

## 1. Introduction

The weakness of the global economy in the post-epidemic era is accompanied by frequent global climate change and secondary environmental problems. The development of traditional fossil fuel energy is facing a potential crisis, and new energy sources offer better environmental compatibility and efficiency [1,2]. Research, development, and application of eco-friendly new energy materials have opened up a broad market. Two-dimensional layered materials are widely used in various fields, which has attracted significant interest and attention in the academic community.

A new family of 2D monolayers, called MXenes, has been discovered. The initial report on MXene-related materials was created by Yury Gogotsi and colleagues at Drexel University in 2011, using HF exfoliation of Ti_3_AlC_2_ [3]. Subsequently, these materials came to be known as MXenes due to their common properties. These monolayers consist of transition metal carbides/nitrides and have the general formula M_i+1_X_i_, where ‘M’ is a transition metal element, ‘X’ is either C or N, and ‘i’ is a positive integer. MXenes are distinguished from traditional 2D materials such as graphene and phosphorene by their higher electronic conductivity, surface hydrophilicity, and unique redox properties. MXenes have unique crystal structures and outer transition metal d-orbital electrons, which differentiate them from traditional materials for catalysis and energy regeneration. MXenes typically exhibit metallic or narrow bandgap semiconductor properties. The material’s band structure and electrons are affected by the outer transition metal [4,5]. As a result, it has a wide range of potential applications in various industrial and commercial fields, including energy storage [6], electrocatalytic reduction [7], photocatalysis [8], renewable energy storage [9], and carbon capture [10]. The field of catalysis has attracted significant attention across various application fields. Electrocatalysis, which includes hydrogen evolution reaction (HER), oxygen evolution reaction (OER), and nitrogen reduction reaction (NRR), is a promising approach to future clean energy conversion technology [11]. MXene can be used as both a catalyst and a carrier in electrocatalysis and is an excellent substitute for precious metal catalysts. Photocatalysis is an advanced catalytic technology that converts solar energy into specific chemical energy. The rate-determining steps of photocatalysis are considered to be the separation and migration of photogenerated charge carriers. To improve the separation efficiency of photogenerated charge carriers, various strategies have been proposed. MXene-based and MXene-derived photocatalysts are rapidly progressing in environmental and energy applications [8,12]. MXene materials typically exhibit high electrical conductivity and specific surface area, making them advantageous for energy storage and conversion applications. Additionally, MXene materials can be doped and surface-modified to achieve even higher performance [13]. Due to their easy-to-produce two-dimensional structure and tunable surface chemistry, MXenes have emerged as excellent materials for carbon capture, which is crucial in mitigating carbon dioxide emissions that contribute to global warming and sea level rise [14].

MAX phase and MXene are catalytic materials that exhibit excellent performance. However, they also have some disadvantages, such as insufficient active reaction sites and unstable catalytic activity [15,16]. Typically, they are integrated into functional composite materials with other substances or doped and modified to enhance their catalytic reaction performance for specific requirements. MXene is often combined with other materials, such as TiO_2_ [17], to enhance photocatalytic efficiency. It can also be doped with Mo and N elements to improve electrode catalytic performance [18,19] or combined with MOF, COF, and other materials for CO_2_ adsorption and capture [20,21]. By improving the thermal stability, electrical conductivity, electrochemical activity, and specific surface structure of the materials, they can become excellent catalysts for photoelectrocatalysis and energy-regeneration reactions. The search for new renewable and alternative energy sources has been triggered by climate change. This has led to the development of new catalytic materials. Feasible strategies for energy regeneration include hydrogen evolution reaction [22], atmospheric nitrogen reduction [23], and carbon capture and conversion to valuable compounds [14]. These strategies can help solve the global energy crisis.

Considering that most previous reviews of MXene have focused on specific applications in the field of catalysis, such as electrochemistry [24], water splitting [25], and energy storage [13], there are still few reports that provide a comprehensive overview of the field of catalysis as a whole. Few articles provide a comprehensive overview of MXene, including a comparison of the advantages and disadvantages of preparation methods, and its performance in various catalytic fields. This paper presents a review of the structural characteristics and common preparation methods of MXene. It also highlights recent advancements in MXene-based catalytic functional materials for electrochemical conversion, photocatalysis, renewable energy, energy storage, and carbon capture and conversion. The article analyses the mechanism of MXene-based photoelectrocatalytic functional materials to enhance catalytic performance and the conversion of high-value compounds. It also points out the difficulties and deficiencies in the field of MXene-based functional materials and looks forward to future development trends and research directions.

## 2. Structural Characteristic and Synthesis Method

### 2.1. Structural Characteristics of MXenes

MXene is a group of materials that comprises transition metal carbides, carbon-nitrides, nitrides, and other related compounds. The structure and composition of MXene are derived from the parent MAX phase. Therefore, the development of MXene has been heavily influenced by MAX phase research. MXene is produced by selectively removing the ‘A’ element from the precursor of layered ternary carbides or nitrides (MAX phases). The chemical formula for MAX phases is M_n+1_X_n_T_x_, where M represents metal atoms such as Ti, Mo, Nb, and V; X represents carbon or nitrogen; *n* can be 1, 2, or 3; and Tx refers to the surface end of -O, -OH, or -F. To etch part A, a fluoride-containing acidic solution is used, and the M layer is capped by a hydrophilic group (-O, -OH, or -F). Examples of MAX phases include Ti_3_C_2_T_x_, Mo_2_CT_x_, and Nb_4_C_3_T_x_, where the M layer covers the X layer in an [MX]_n_M arrangement [26]. These materials have unique advantages, including excellent conductivity, a large surface area, a tunable structure, and hydrophilicity. In addition to the conventional ternary MAX phase, alloying of M, A, or X sites is crucial from both an application and scientific point of view. The addition of other elements can adjust the properties and form unconventional MAX phases. Alloying of MAX phases typically results in chemically disordered solid solutions. However, MAX phase alloys can also exhibit chemically ordered structures. Two types of chemical ordering have been observed in MAX phases: out-of-plane chemical ordering (oMAX) and in-plane chemical ordering (i-MAX), both with M elemental ordering [27].

**Figure 1:**
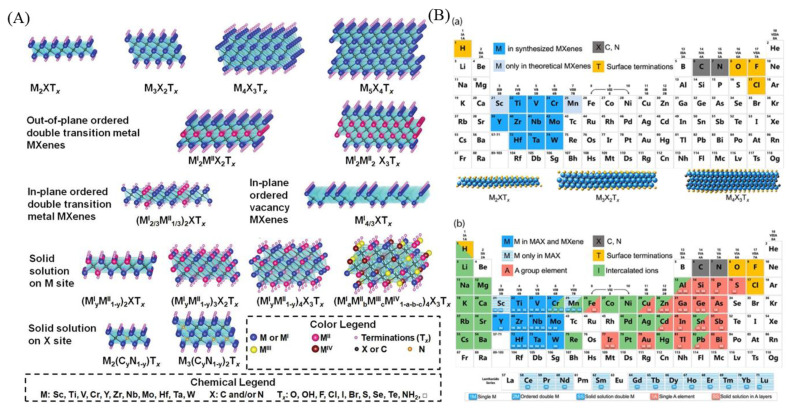
(**A**) Atomic models of different known MXene structures [28]. (**B**) Periodic table of elements composed of MXenes and MAX. (**a**) Elements used to build MXene. (**b**) Elements used to build the MAX phase, MXene, and their intercalated ions [29].

Figure 1A displays the known atomic models of various MXene structures. MXenes have a molecular formula of M_n+1_X_n_T_x_, where M is a transition metal, X is either C or N, Tx represents the surface terminal, and *n* ranges from 1 to 4. The six major types of MXene structural models are single transition metal doping, in-plane and out-of-plane dual metal doping, vacancy type, and solid solvent type at M and X sites [28]. Figure 1B displays the periodic table of elements composed of MXenes and MAX in a clear and concise manner. The four background colors in the figure represent different components of MXene. Dark blue represents an element that has been used to synthesize MXenes, light blue represents a theoretically possible element, and yellow represents the surface terminal element. Figure (b) in Figure 1B shows that the dark blue elements are present in both the precursor and MXene, while the blue shading is only present in the precursor. The green elements are reported for doping ions [29].

### 2.2. Methods for Synthesising MXenes

**Figure 2:**
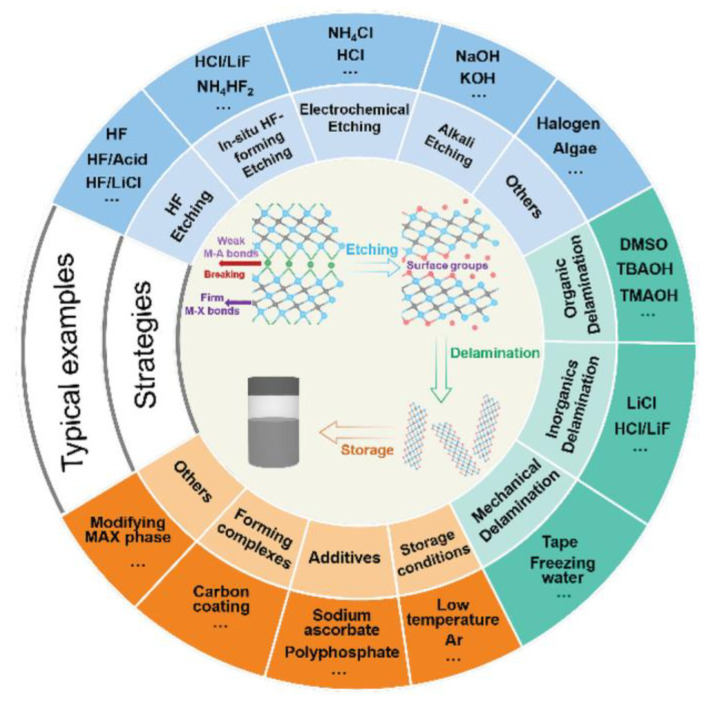
Overview of reported etching methods, layering strategies, and storage methods for MXene synthesis [30].

Different synthesis methods have their own advantages, disadvantages, and key influencing factors (Table 1). The synthesis strategy is to selectively remove the middle A layer to form a specific two-dimensional structure, taking into account the fact that the M–A bond in MAX is relatively fragile and easily broken, while the relatively strong M–X bond is protected [29]. Figure 2 provides an overview of current strategies and reagents related to MXenes synthesis [30]. This section mainly introduces acid etching, fluoride salt etching, and electrochemical etching. In the future, large-scale preparation and industrial applications will require synthetic strategies that are cost-effective, efficient, and produce high-quality results [31,32].

In 2011, MXene was first prepared through HF etching. It was discovered that HF can selectively etch the middle atomic layer in Ti_3_AlC_2_, forming an accordion-shaped product with layers connected by van der Waals forces [3]. Classical HF etching can speed up the etching and increase the reaction rate at high concentrations. However, this concentration effect can also increase the defects of resulting Ti_3_C_2_T_x_ nanosheets and damage their structure. As a result, the physicochemical properties and performance of the nanosheets in photovoltaics and catalysis may be reduced. It is important to balance the concentration to avoid these negative effects [33]. The structure and properties of MXenes are significantly affected by the etchant used. Hydrofluoric acid etchant is capable of effectively etching most Al-containing MAX phases. However, excessive amounts of HF can result in more defects. Mixing the HF etchant with another acid, such as HCl or H_2_SO_4_, can increase the efficiency of the etch and reduce the reliance on a single acid [34]. Anayee et al. [35] investigated the etching of the Ti_3_AlC_2_ MAX phase using a concentration of 5 wt.% HF and different acid mixtures, namely HF/HCl and HF/H_2_SO_4_. The material was also studied in the multilayer stage to prevent changes during delamination. The results indicate that etching with HF/H_2_SO_4_ yields higher conductivity and thermal stability values compared to other etching methods. Studies on electrochemical sodium ion intercalation reveal that mixed-acid etching produces a cleaner surface by effectively removing etch by-products and structural water, leading to improved electrochemical properties and cyclability.

Fluoride salt etching is a method that uses fluoride salts and acids as etchants to generate hydrofluoric acid (HF) to prepare MXenes in situ. The etching process involves the insertion of ions into the accordion-shaped Ti_3_C_2_T_x_ layer to expand the layer spacing. Subsequent ultrasonic or oscillation treatment can directly convert the layered material into single-layer/few-layer nanosheets. Numerous studies have reported the feasibility and advantages of fluoride salt etching preparation. Wang et al. [36] utilized controlled hydrochloric acid-assisted hydrothermal etching to generate established surface functional groups. The resulting Mo_2_C electrode exhibited high electrochemical performance in supercapacitors. Density functional theory calculations were employed to demonstrate the etching feasibility of various MAX phases with hydrochloric acid (HCl). The findings indicate that several MAX phases can be effectively etched in HCl solution alone through the formation of ACl_3_ (A = Al, Ga). Simultaneously, pressure and temperature are crucial factors in the etching process of TRL, which enables the industrial production of fluorine-free MXene. Liu et al. [37] prepared two-dimensional MXene Ti_3_C_2_ and Ti_2_C structures from Ti_3_AlC_2_ and Ti_2_AlC by etching LiF, NaF, KF, and NH_4_F with HCl. These structures were then used to capture methane or toxic and harmful gases. MXenes made of NaF and KF can absorb methane under high pressure and release it at low pressure, which has the potential for use in the adsorption storage of natural gas.

Electrochemical etching, using MAX as the electrode, is a method for removing the middle Al atomic layer under specific voltage. This method is an alternative to the classic acid etching method. The removal rates and product structures vary depending on the electrolyte used. It is important to note that this method has different removal rates and product structures depending on the electrolyte used. Selective etching of MAX can be accurately controlled by adjusting the voltage window and potential. The rate of electrochemical etching and the structural characteristics of the product are affected by different electrode voltages in relation to the acid concentration. MXene will be prepared through electrochemical one-pot etching, resulting in the production of Cl-terminated Ti_2_C MXene (Ti_2_CCl_x_) from Ti, Al, and C powders in molten salt LiCl/KCl [38]. The surface structure and properties will be precisely controlled by adjusting carbon sources such as CNT and rGO, which will serve as the corresponding carbon template. Yin et al. [39] developed a straightforward electrochemical etching method to synthesize Ti_3_C_2_F_x_ and control the degree of fluorination. The electrolyte consists of a room-temperature ionic liquid (RTIL) [BMIM] [PF_6_] and MeCN. Non-aqueous etching effectively prevents MXene oxidation during the process. The MXene product exhibits controllable fluorination and excellent electrochemical activity. At a current density of 200 mA g^−1^, it has an initial charge capacity of 329 mA h g^−1^, which is maintained at 211 mA h g^−1^ after 500 cycles. This feature makes it a valuable asset in the design and development of superior batteries.

**Table 1:** Comparison of typical MXene synthesis methods.

Strategies	Advantages	Disadvantages	Influencing Factors	Ref.
Acid etching	Easy operation and low reaction temperature.	High corrosiveness, toxicity, and ecological and environmental risks.	Etchant concentration, etching temperature, and etching time.	[40]
Alkali etching	High etching efficiency and low impurity content.	Strong alkali and high temperatures in reaction conditions cause safety hazards.	The alkali concentration and reaction temperature.	[41]
Fluoride salt etching	Mild conditions and safe preparation.	Relatively high impurity content and low yield.	Acidity of organic anions and concentration of dissociated fluoride ions.	[30]
Electrochemical etching	Less acidic etchant content, mild conditions, and low energy consumption.	Low productivity and additional equipment costs.	Etching voltage window (etching potential) and etching time.	[42]
Molten salt etching	Mild reaction conditions and wide etching range.	The product structure has poor stability and the etching efficiency is unstable.	The type and concentration of molten salt.	[43]

## 3. Catalytic Applications

### 3.1. Electrocatalysis

#### 3.1.1. Hydrogen Evolution Reactions (HER)

**Figure 3:**
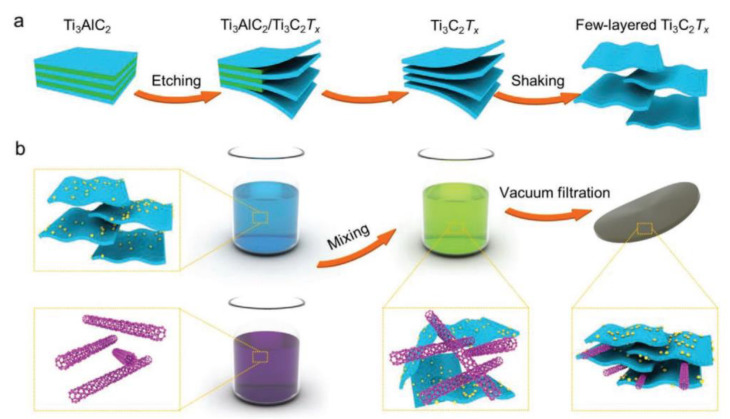
Mechanism diagram: (**a**) Schematic diagram of Ti_3_C_2_T_x_ MXene colloidal suspension and (**b**) Design of MXene@Pt/SWCNTs nanocatalyst [49].

Directing water electrolysis using electricity generated by clean/sustainable energy and solar energy will be a promising technology for producing renewable energy in the future [44,45,46]. The entire process of clean energy production must ensure environmental friendliness, and hydrogen (H_2_) has the potential to be a clean energy source. The energy density is as high as 120–140 MJ kg^−1^, making it a green and efficient energy source [46]. The hydrogen evolution reaction (HER) is an efficient way to convert electricity. Traditional Pt electrodes cause energy loss and consumption. Considering the cost and availability of precious metals, new environmentally friendly, efficient, and energy-saving HER catalysts and electrode materials are of great interest [47,48]. Cui et al. [49] proposed a system to construct a hierarchical Pt–MXene–single-walled carbon nanotube (SWCNT) heterostructure for HER catalysts. Figure 3 shows the synthesis strategy. Initially, Ti_3_AlC_2_ is etched in a solution containing hydrochloric acid and ethanol to obtain Ti_3_C_2_T_x_ MXene flakes. These flakes are then treated twice in a colloidal suspension. The two suspensions are mixed to form a stable suspension, and finally, the Ti_3_C_2_T_x_@Pt/SWCNT material membrane is prepared through vacuum filtration. Layered MXene@Pt/SWCNTs were utilized to retain their physicochemical properties after the reduction of platinum complex ions and SWCNTs acting as conductive binders, resulting in high-performance HER catalysts. The catalysts exhibited a high-volume current density of up to 230 mA cm^−3^ at −50 mV and low overpotential compared to the reversible hydrogen electrode (RHE). At a current density of −10 mA∙cm^−2^, the voltage relative to RHE was −62 mV. The stratified HER catalyst demonstrated excellent stability over ten days of operation.

**Figure 4:**
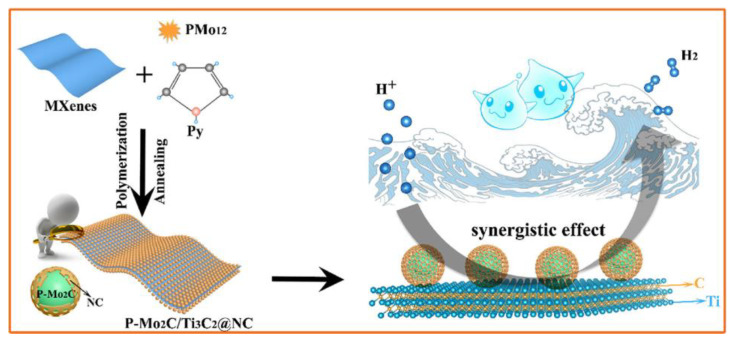
Synthesis strategy: Schematic diagram of P-doped molybdenum carbide nanodots and synergistic effects on nitrogen-doped carbon shell-encapsulated Ti_3_C_2_ flakes [51].

The use of non-noble electrocatalytic materials for hydrogen production in alkaline media is a cost-effective strategy. The Ni_2_P/Ti_3_C_2_T_x_/NF electrocatalyst material, designed with a unique structure and coupled synergistic effect of Ti_3_C_2_T_X_ and Ni_2_P, produces a current density of 10 mA∙cm^−2^ at a low overpotential (135 mV) with excellent stability and efficiency in an alkaline environment [50]. An article reported a new nanohybrid material consisting of phosphorus-doped molybdenum carbide nanodots supported on nitrogen-doped carbon-encapsulated Ti_3_C_2_ flakes [51]. The P-Mo_2_C/Ti_3_C_2_@NC nanohybrid material is composed of ultra-small P-doped Mo_2_C nanodots that are uniformly anchored on Ti_3_C_2_ flakes encapsulated in nitrogen-doped carbon shells. Figure 4 shows that in the preparation mechanism, ultra-small P-doped Mo_2_C nanodots are uniformly anchored on nitrogen-doped carbon shell-encapsulated Ti_3_C_2_ flakes to form a P-Mo_2_C/Ti_3_C_2_@NC 2D nanohybrid through a simple two-step route of polymerization and annealing. P-doped Mo_2_C is obtained using phosphomolybdic acid (H_3_PMo_12_O_40_, PMo_12_) as the molybdenum source, and pyrrole (Py) polymerization is initiated. After annealing, polypyrrole (PPy) forms a nitrogen-doped carbon shell that covers the surface of the composite. The combined effects of polymerization and annealing have successfully produced P-Mo_2_C/Ti_3_C_2_@NC nanohybrid materials. The P-Mo_2_C/Ti_3_C_2_@NC nanohybrid catalyst exhibits remarkable performance, with HER activity of 177 mV at 10 mA∙cm^−2^, fast reaction kinetics of 57.3 mVdec^−1^, and good long-term stability in acidic electrolytes. The excellent performance of the HER may be attributed to the synergistic coupling and multifunctional interfacial catalytic interaction between P-doped Mo_2_C, N-doped porous carbon, and Ti_3_C_2_ components.

Precise modulation of the hydrogen extraction reaction (HER) activity of MXene using vacancy engineering is another way to broaden the energy of MXene. H adsorption is either too strong or too weak, and a single C vacancy will slightly weaken H adsorption without significantly altering the intrinsically high activity, whereas a double C vacancy can effectively weaken binding and promote activity [52]. Electronic structure analyses show that the introduction of C and M vacancies triggers the upward and downward shifts of the Fermi energy levels of the surface oxygen into the conduction and valence bands, respectively, with corresponding weakening and strengthening of the O-H interactions. The activity modulation correlates with the electronic structure of the defective MXene, where the highest occupied peak position of the surface O electronic state shows a clear linear trend with ΔG_H_ [52]. Defect chemistry and vacancy engineering have the potential to modulate the catalytic activity of MXene, which is conducive to further enhancing the activity of the composite.

#### 3.1.2. Nitrogen Reduction Reaction (NRR)

NH_3_ is a crucial chemical raw material used extensively in chemical, pharmaceutical, and petroleum industries [53,54,55]. Nitrogen elements in the natural environment are mostly present in the form of nitrogen in the air and are not effectively utilized. Traditional nitrogen fixation methods have several drawbacks, including high energy consumption, severe environmental pollution, and low efficiency [56,57]. Efficient and clean conversion of nitrogen into valuable industrial products is a widely researched topic. Electrochemical methods are commonly used in NRR reactions due to their controllability and stability. The use of MXene materials in NRR reactions is a promising development direction [58,59]. NRR technology is an effective means to mitigate environmental hazards and track greenhouse gas emission footprints in the NH_3_ production process. Johnson et al. [60] synthesized Ti_2_N nitride MXene using oxygen-assisted molten salt fluoride synthesis technology. They utilized the Mars–van Krevelen (MvK) mechanism on the two-dimensional (2D) Ti_2_N nitride MXene to achieve a low voltage of −250 mV relative to RHE. The applied potential resulted in a yield of 11.33 μg/cm^2^/hr, and the FE for NH_3_ was 19.85%.

Due to the slow dissociation of the NN triple bond, the electrochemical nitrogen reduction reaction (NRR) typically has relatively low Faradaic efficiency and ammonia yield. Single-atom catalysis is a current research focus. Peng et al. [61] reported that Ru atoms doped with rich Mo defective sites can chemically activate MXene for high-performance NRR catalysis under environmental conditions. At −0.3 V, the catalyst exhibited a Faradaic efficiency of 25.77% and an ammonia yield of 40.57 µg h^−1^mg^−1^ when compared to a reversible hydrogen electrode in a 0.5 M K_2_SO_4_ solution. The calculations indicate that Ru anchored on MXene nanosheets serves as a crucial feedback center for catalysis, facilitating nitrogen adsorption and activation. In another study on single-atom NRR, Fang et al. [62] supported Fe and Ru single atoms on MXene (Nb_2_C) to form an excellent NRR electrocatalyst, Ru/Nb_2_CO_2_. The selectivity of Ru/Nb_2_CO_2_ in generating *NNH is as high as 99.9%, which is highly beneficial for the catalytic synthesis of ammonia. The composite exhibits good thermal stability in a 10 ps AIMD simulation at 500 K. Guo et al. [63] improved the NRR catalytic reaction activity by introducing Fe to the MXene surface and calcining it to eliminate the inactive surface F*/OH* terminals, exposing more active sites. The best-performing catalyst (MXene/TiFeO_x_-700) achieved a Faradaic efficiency of 25.44% and an NH3 yield of 2.19 µg/cm^2^·h.

**Figure 5:**
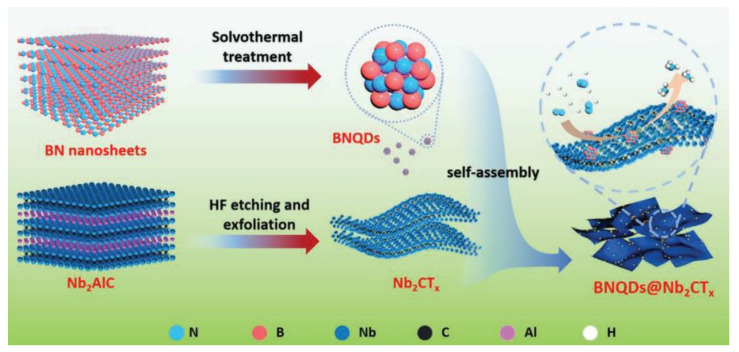
Schematic of the synthesis process of BNQDs@Nb_2_CT_x_ [65].

Doping MXene with heteroatoms is a common method for enhancing electrochemical performance. In this study, 3D N-doped-Ti_V_-Ti_3−x_C_2_T_y−1.2_ MXene was produced through ENRR. Ti^3+^ serves as the active site for electrocatalytic nitrogen reduction reaction (ENRR) [64]. Its electronic state can be adjusted through surface atom engineering to create vacancies and doping. The introduction of Ti vacancies can capture electrons injected into the N_2_ antibonding orbital, which is beneficial for the activation of N_2_. The addition of a dopant reduces the orbital overlap between N_2_ and Ti^3+^ and lowers the reaction energy barrier, resulting in a beneficial effect on the desorption of NH_3_. The composite material, consisting of the new Nb_2_CT_x_-MXene (BNQDs@Nb_2_CT_x_) and hexagonal boron nitride quantum dots (BNQDs), exhibits excellent NRR activity. At −0.4 V, it yields 66.3 µg h^−1^ mg^−1^ of NH_3_ with a Faradaic efficiency of 16.7%. The material also demonstrates good electrical stability. The addition of quantum dots enhances the activation of N_2_ and inhibits the generation of H_2_ [65]. Figure 5 illustrates the synthesis mechanism of BNQDs@Nb_2_CT_x_. BNQDs quantum dots are obtained through solvothermal BN nanosheets, which are supported on MXene nanosheets formed by HF etching. The self-assembly process results in a BNQD@Nb_2_CT_x_ composite material that serves as an NRR catalyst.

**Figure 6:**
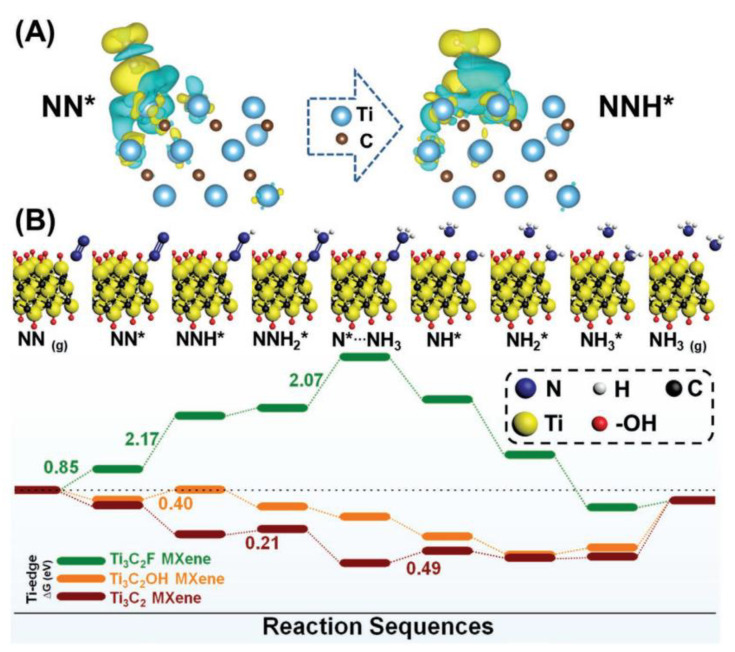
Ti_3_C_2_OH MXene composite reaction mechanism diagram and free energy scheme. (**A**) Optimized charge density difference at the surface edge of Ti_3_C_2_OH MXene. (**B**) Reaction mechanism diagram and free energy scheme on edge Ti [66].

In another study on quantum dot doping, hydroxyl-rich Ti_3_C_2_T_X_ QDs (Ti_3_C_2_OH QDs) were synthesized through alkalization and intercalation as NRR catalysts. The material exhibited a NH_3_ yield of 62.94 µg h^−1^mg^−1^_cat_ [66]. MXene catalysts can efficiently regulate ammonia production at room temperature by optimizing QD size and functional groups. Ti_3_C_2_OH QD exhibits good catalytic activity, selectivity, and stability. Figure 6 shows that charges of NN* and NNH* are transferred from N_2_ to the Ti edge on the composite surface, and nitrogen molecules are attached to the Ti edge, which may serve as good catalytic active sites. The data indicates that the N_2_ adsorption reaction on Ti_3_C_2_F has a higher potential energy, while the N_2_ adsorption reaction of the other two materials is exothermic and spontaneous. A summary of typical examples of MXenes-based composite electrocatalysts will be shown in Table 2.

### 3.2. Photocatalysis

In the field of photocatalytic hydrogen precipitation, TiO_2_ has carried out a large number of reported studies due to its abundance, low price, effectiveness, and suitable CB and VB edge energy levels [78,79,80,81]. However, the existence of traditional TiO_2_ has the disadvantages of low light utilization and fast photogenerated carrier complexation hindering its further industrial large-scale application [82,83]. The electronic properties of MXenes are related to end groups and transition metal elements. Different materials with end groups or transition metals can be transformed into semiconductors or topological insulators. The conductivity of MXenes is closely related to the morphology of the preparation process as well as the etching and deposition conditions. For example, when the synthesis conditions are changed, the conductivity of Ti_3_C_2_TX also changes [84]. Interlayer contacts and intercalated species can influence the electrical conductivity of MXenes. For example, conductivity can be affected by surface functional groups that replace the ‘A-element’ layer, as well as the presence of intercalated TBA and water molecules between MXene nanosheets. Annealing the MXene paper at 800 K (top pattern) causes the (002) peak to shift to a higher angle due to the insertion of the OH termination and other end groups [85]. Therefore, the combination of TiO_2_ and MXene for photocatalytic applications has become a hot research topic nowadays [17,86,87] (Table 3).

To enhance the catalytic activity of photocatalytic degradation of pollutants, metal doping and functional material composite strategies are commonly employed. Han et al. [87] designed a precursor for the preparation of C-TiO_2_/g-C_3_N_4_ photocatalysts without the addition of additional carbon, and the highest photocatalytic hydrogen production activity of C-TiO_2_/g-C_3_N_4_ photocatalysts was 1409 μmol/h/g at the mass ratio of Ti_3_C_2_ to g-C_3_N_4_ of 10 wt.%. The possible mechanism is that a close heterojunction between the Ti_3_C_2_ MXene-derived C-doped TiO_2_ and g-C_3_N_4_ can effectively promote photogenerated charge transfer and inhibit electron and hole complexation, thus significantly enhancing the photocatalytic hydrogen production activity. Wu et al. [88] demonstrated the active decomposition of pollutants by anchoring a graphene layer on a TiO_2_/g-C_3_N_4_ high visible light active photocatalyst for the purification and degradation of water pollutants, benefited by the synergistic effect between graphene, TiO_2_, and g-C_3_N_4_. The main species ∙O_2_^−^ and ∙OH involved in the active degradation of pollutants were confirmed by radical capture and electron spin resonance experiments. GTOCN_3_ showed excellent visible photocatalytic synergistic degradation of TC (0.02442 min^−1^), CIP (0.01675 min^−1^), BPA (0.01935 min^−1^), and RhB (0.05586 min^−1^). MXene is proven to be an excellent cocatalyst, accelerating charge separation and suppressing exciton recombination. In terms of the photocatalytic degradation of environmental pollutants, the doping amount of MXene affects the photocatalytic degradation efficiency of TiO_2_-MXene composite materials. The researchers found that 5 wt.% MXene has the best photocatalytic activity for benzene degradation while continuing to increase the proportion leads to a decrease in photocatalytic activity [89]. Under visible light irradiation, ZnO nanorods/MXene (ZnO-MX) hybrids prepared by ultrasonic oscillation exhibited higher rhodamine B (RhB) elimination than pure ZnO nanorods, with a removal efficiency 3.2 times higher than the latter [90]. The 1 wt.% MXene/g-C_3_N_4_ heterostructured photocatalysts synthesized by wet impregnation achieved efficient degradation of methylene blue under 180 min of visible light irradiation [91]. The introduction of MXene further improved the charge separation and transport and efficiently facilitated the degradation of the dye in wastewater under solar radiation. Zakarya et al. [92] prepared transition metal nanomaterials decorated with MXene nanocomposites for the photocatalytic degradation of methylene blue (MB) and rhodamine B (RhB) by a facile one-pot hydrothermal method. The structure of the nanocomposites confirmed the stable formation of MNPs/TiO_2_/Ti_3_C_2_T_x_ and the nucleation of these TiO_2_ particles on the MXene surface. The excellent performance of AgNPs/TiO_2_/Ti_3_C_2_T_x_ photocatalysts was attributed to the positive effects of anatase TiO_2_ and silver particles in enhancing the light trapping, dye adsorption, and charge separation in the treatment of total organic carbon was reduced by 23% after treatment of industrial wastewater.

In the study of photocatalytic hydrogen precipitation reaction, the hydrogen precipitation performance and reaction stability were improved by the addition of MXene-oriented tuned heterostructured photocatalysts. Zuo et al. [93] used sandwich-like layered heterostructured (UZNs-MNs-UZNs) MXene nanosheets for efficient photocatalytic hydrogen precipitation. The ultrathin two-dimensional structure of ZNs and MNs effectively inhibited the stacking and agglomeration of active sites and promoted photogenerated charge transfer and segregation, and the composites had 6.6 times the hydrogen precipitation performance of the pristine materials. A two-step hydrothermal method was used to construct a new CdS/MoO_2_@Mo_2_C-MXene photocatalyst for hydrogen production. Its relatively narrow band gap is conducive to visible light absorption. The CdS/MoO_2_@Mo_2_C-MXene composite catalyzed hydrogen production reaches 22,672 μmol/(g·h), 11.8 times that of pure CdS. The results show that the binary cocatalyst has higher photocatalytic hydrogen production activity than the single cocatalyst Mo_2_C MXene [94]. Peng et al. [95] used a hydrothermal method to grow Nb_2_O_5_ nanorods perpendicular to the basal surface of Nb_2_CT_x_. They used a layered heterostructure to design an Ag/Nb_2_O_5_@Nb_2_CT_x_ hybrid to overcome the disadvantages of rapid recombination of photogenerated electrons and holes. Using glycerol as a sacrificial agent, the HER activity reached 824.2 μmol⋅g^−1^h^−1^.

**Table 3:** Summary of typical examples of MXenes-based composite photocatalysts.

Photocatalysts	MXenes	Catalytic Activity	Ref.
C-TiO_2_/g-C_3_N_4_	Ti_3_C_2_	Photocatalytic hydrogen production activity was 1409 μmol/h/g.	[87]
TiO_2_/graphene/g-C_3_N_4_	Ti_3_C_2_	The degradation rate was TC (0.02442 min^−1^), CIP (0.01675 min^−1^), BPA (0.01935 min^−1^), and RhB (0.05586 min^−1^).	[88]
Ag/Nb_2_O_5_@Nb_2_CT_x_	Nb_2_CT_x_	HER activities were 682.2 and 824.2 μmol⋅g^−1^h^−1^, respectively.	[95]
MoS_2_/TiO_2_/Ti_3_C_2_	Ti_3_C_2_	The optimum H_2_ evolution rate of 6425.297 μmol/h/g was obtained on	[96]
CdLa_2_S_4_/Ti_3_C_2_	Ti_3_C_2_	The maximum hydrogen production rate was 11,182.4 μmol/h/g.	[97]
Ag_3_PO_4_/Ti_3_C_2_	Ti_3_C_2_	The photocatalytic performance toward tetracycline hydrochloride was 68.4%.	[98]
TiO_2_/Ti_3_C_2_	Ti_3_C_2_	The photocatalytic H_2_ production rate was 218.85 μmol g^−1^ h^−1^.	[99]
CdS/Ti_3_C	Ti_3_C_2_	Visible light photocatalytic hydrogen production activity was 14,342 μmol h^−1^g^−1^.	[100]
Mo_x_S@TiO_2_@Ti_3_C_2_	Ti_3_C_2_	The hydrogen production from photocatalytic water decomposition is 10,505.8 μmol g^−1^h^−1^.	[101]
Ti_2_C/3%TiO_2_/1%Ag	Ti_2_C	Salicylic acid (SA) photodegradation was 86.1–97.1% within 3 h; SA initial solution concentration was 100 μM.	[102]
Bi_2_WO_6_/Nb_2_CT	Nb_2_CT_x_	The degradation efficiencies of the photocatalysts were 99.8%, 92.7%, and 83.1% for RhB, MB, and TC-HCl, respectively.	[103]
MXene/ZnIn_2_S_4_	Ti_3_C_2_T_x_	Within 45 min under simulated visible light irradiation, the Cr(VI) reduction and MO degradation rates were as high as 93.4% and 96.9%, respectively.	[104]

### 3.3. Renewable Energy and Energy Storage

The economy’s rapid development is heavily reliant on energy. Due to the numerous drawbacks of traditional fuels, there has been a significant focus on green and sustainable energy. Visible energy can be converted into electrical energy and stored in an energy storage device, with rechargeable batteries being an effective and important option [105]. Two-dimensional MXenes have great potential in the field of rechargeable batteries, particularly as a substrate for the in situ growth of various materials [106]. Functional materials can be grown in situ on two-dimensional DMXene through various strategies. Thanks to the special advantages of 2D MXene, in situ grown MXene-based composite materials have been widely used in rechargeable battery components [107] (Table 4). Liao et al. [108] developed a heteroatom doping strategy to precisely modulate the surface functionality of MXene, and the combination of S- and N-doped MXene with large rGO sheets allowed the fabrication of large-area and environmentally stable MXene-based films (SNMG-40) with high mechanical strength (45 MPa) and energy storage (698.5 F cm^−3^) by squeegee deposition. SNMG-40 films exhibited good cyclic stability and were resistant to environmental influences while aMGSC showed an ultra-high energy density of 22.3 Wh kg^−1^.

As a sustainable green energy, solar energy will be an efficient and environmentally friendly means of powering the thermal energy storage and conversion of MXene materials. Studies have shown that the extinction coefficient of MXene nanosheets at 808 nm in MXene aerogel-based high-performance phase change materials is 25.67 L/(g∙cm), showing excellent light absorption properties. Aerogel-based functional composites composed of MXene nanosheets and PEG were prepared by freeze-drying, which can effectively absorb solar energy and convert it into thermal energy for storage. The photothermal storage efficiency of MXene@PEG aerogel reaches 92.5%. The introduction of a porous structure reduces the overall density of the material and stabilizes the material structure [109]. Gong et al. [110] successfully prepared SSPCM based on polyurethane/MXene with thermal stability and photothermal effect, in which MXene serves as a photothermal conversion enhancer, and the functional composite has excellent solar-thermal conversion efficiency and mechanical stability. The melting enthalpy and crystallization enthalpy of 2.0-MPH reach 127.97 J/g and 119.32 J/g, respectively. The additional introduction of MXene significantly improves the photothermal conversion capability and thermal stability of the material. The photothermal conversion efficiency of 2.0-MPH is as high as 90.45%, which has broad commercial application prospects. By chemically modifying phosphorus-containing molecules, the corresponding flame-retardant phase materials prepared have good thermal conductivity and photoelectric storage capabilities. Porous MXene serves as the supporting framework, and the prepared MXene-based PCM (PSM-4) has good thermal conductivity (0.486 W m^−1^K^−1^) and maintains shape stability when heated to high temperatures [111]. As the MXene loading in the composite increases, the pHRR and THR values of PCM decrease, making it an excellent solar energy storage and flame retardant material.

**Figure 7:**
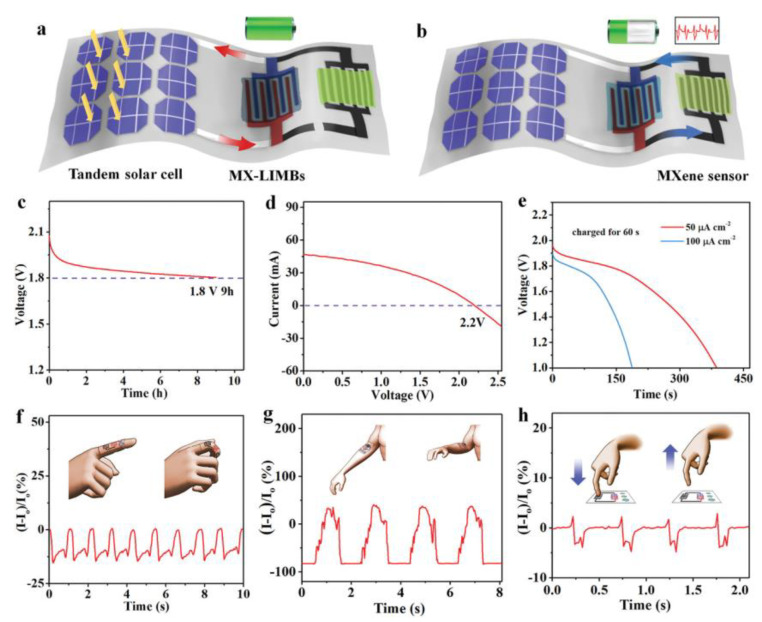
Current effect and self-powered integrated system for pressure sensing based on MXene. (**a**) Schematic of the process for charging by the flexible tandem Si-SC. (**b**) Schematic of the process with the sensor powered by the MX-LIMB. (**c**) Self-discharge profile of the MX-LIMB. (**d**) *I–V* curve of the flexible tandem Si-SC on the stainless-steel substrate. (**e**) Discharge curves of the MX-LIMB at different current densities after charging by the tandem Si-SC for 60 s. (**f**–**h**) Current change of the MXene hydrogel sensor powered by the integrated MX-LIMBs in response to the bending of a finger (**f**), the bending of an elbow (**g**), and pressing vertically (**h**) [112].

In terms of energy conversion and storage of MXene for wearable devices, the combination of different functional components will expand the sustainable use of health monitoring devices. Water-based additive-free MXene inks and MXene-based battery-based inks designed by Zheng et al. enable rapid and scalable fabrication of MX-MSCs and MX-LIMBs with customized shapes on insulating substrates. The MSCs exhibit an ultrahigh area capacitance of 1.1 F cm^−2^, and the MSCs in series provide a record voltage of 60 V [112]. Integrated systems with composite architecture that are sensitive to body responses are expected to be used in future smart wearable devices. Figure 7 shows the MXene-based integrated system. Figure 7a shows the schematic diagram of the charging process of MX-ILMBs, and Figure 7b shows the energy dissipation process in operation for the sensors. Figure 7c–e shows the discharge graphs and current and voltage curves. Figure 7f–h vividly illustrates the corresponding current variations triggered by the body movements such as finger, arm, and fingertip presses. The self-powered integrated system consists of an energy harvester, energizer, and water pressure sensor and is capable of self-running on a solar energy supply [112]. In flexible asymmetric capacitor applications, the preparation and selection of suitable cathode and anode materials are decisive factors. The Ti_3_C_2_T_x_ MXene//V_2_O_5_ asymmetric flexible energy storage device assembled by Qian et al. [113] has good matching with the anode material. The MXene layered sheet structure helps the anode and cathode electrodes operate under the potential windows of −1.1 to 0.1 and −0.1 to 0.4 V, respectively. Achieving ideal capacitive performance and providing an energy density of 8.33 mWh cm^−3^ at a current density of 0.5 A g^−1^, the composite material can be used in high energy density flexible energy storage devices.

**Table 4:** Summary of typical examples of MXenes-based energy storage catalysts.

Catalysts	MXenes	Catalytic Activity	Ref.
Ti_3_C_2_T_x_/C	Ti_3_C_2_T_x_	The supercapacitor electrode exhibits a high specific capacity of 226 F g^−1^ at 1 A g^−1^ with 94% retention over 8000 cycles.	[114]
Graphite/ MXene	Ti_3_C_2_	A high energy density of over 80 mW h/g and a rate capability of 75 mW h/g, with a capacity fading of 5% after 1500 cycles.	[115]
IL-MXene	Ti_3_C_2_	The thermal conductivity is 0.82 W/m·K at 20 °C, and the specific capacity of pure IL aqueous solution is 2.374 J/g K.	[116]
V_2_CT_x_	V_2_CT_x_	With a gravimetric capacitance of 900 F g^−1^, the intercalated electrode exhibits excellent Coulombic efficiency (100%) for 10,000 GCD cycles at a current density of 2 A/g.	[117]
SSPCMs	Ti_3_C_2_T_x_	The melting phase change enthalpy and relative enthalpy efficiency are 127.97 J/g and 76.96%, respectively, while the photothermal conversion efficiency (θ) is 90.45%.	[110]
PCM/MXene	Ti_3_C_2_	Compared with pure paraffin, the absorbance of the composite increased by 39% and the maximum thermal conductivity increment was 16%.	[118]
MXene/EUPCM (1:99)	Ti_3_C_2_	The addition of MXene resulted in a consistent reduction in heating and cooling times.	[119]
MoS_2_@MXene	Ti_3_C_2_	The maximum electric displacement at room temperature is 10.96 μC/cm^2^, and the discharge energy density reaches 17.22 J/cm^3^.	[120]
MXene/MgCr_2_O_4_	Ti_3_C_2_T_x_	The maximum capacitance value observed in alkaline media is 542.6 F/g, while the minimum capacitance value is 454.1 F/g in acidic media.	[121]
MXene-C60	Ti_3_SiC_2_	The highest capacitance of MXene-C60 composite is 348 F g^−1^.	[122]
Paraffin/Ti_3_C_2_T_x_@gelatin	Ti_3_C_2_T_x_	The composite material has a high loading ratio (96.3–97.7%) and large melting enthalpy (184.7–199.9 J/g).	[123]
N-Ti_3_C_2_T_x_-300	Ti_3_C_2_T_x_	Provides a maximum volumetric energy density of 21.0 Wh L^−1^ and an energy density of 10.2 Wh L^−1^ at a high power density of 18.3 kW L^−1^.	[124]
SMPCCs	Ti_3_C_2_	The phase change material maintained up to 93 wt.% PEG loading without any leakage, with a relative thermal efficiency loss of only 1% after 100 heating-cooling cycles.	[125]

### 3.4. Carbon Capture and Conversion

The combustion of fossil fuels emits a significant amount of greenhouse gases, with carbon dioxide being particularly important. Researchers worldwide are working to reduce carbon dioxide emissions to mitigate the greenhouse effect. Two crucial areas of development are the creation of cleaner energy and the development of efficient carbon dioxide capture materials [126,127,128]. The use of transition metal carbides as materials for capturing CO_2_ has been extensively studied (Table 5). MXene, a family of layered nanosheets with uniformly terminated functional groups and oxygen vacancies, is a promising support for CO_2_ adsorption due to its high mechanical strength, low heat capacity, and high thermal conductivity [129,130,131,132].

**Figure 8:**
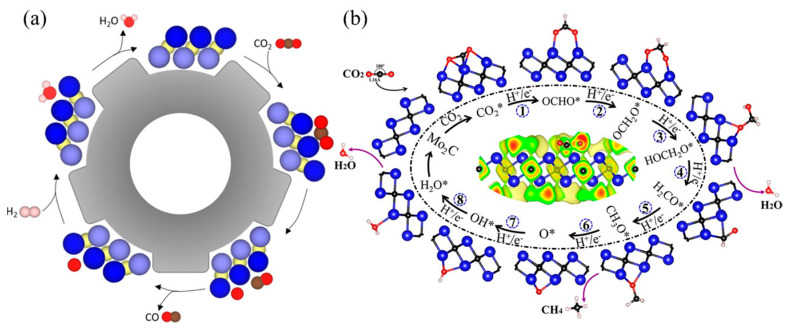
(**a**) Scheme of the steps carried out for the RWGS reaction. Dark blue spheres and light blue spheres represent metal atoms in the upper and lower layers of MXene respectively, yellow spheres represent X (C or N) atoms, red represents oxygen atoms, brown represents carbon atoms, and pink represents hydrogen atoms [128]. (**b**) Structural diagram of the energy pathway of Mo_2_C MXene catalyzing the reduction of CO_2_ to CH_4_ [130].

Raul et al. [128] investigated the CO_2_ dissociation equilibrium of 18 carbide and nitride MXene catalysts using DFT calculations and kinetic phase diagrams. They found that in RWG catalysis, which requires a hydrogenation step for regeneration, the MXene surface charge and d-band centers are crucial for reactant adsorption (Figure 8a). The researchers also discovered that the MXene surface becomes covered with O atoms, necessitating a catalyst regeneration step that involves annealing and surface O hydrogenation to generate and desorb H_2_O. MXenes are suitable for capturing carbon dioxide and converting it into economically valuable products, promoting renewable and sustainable carbon closed cycles. Guo et al. [130] conducted a systematic study on the application of M_2_C-type MXenes in CO_2_ capture and conversion using density functional theory. The study found that MXene has a better ability to capture CO_2_ molecules than H_2_O molecules, and the captured CO_2_ can be further reduced to CH_4_ under the activation of M_2_C MXene. Notably, Group VI Mo_2_C MXene exhibited superior catalytic performance. All M_2_C-type MXenes exhibit spontaneous CO_2_ capture and activation capabilities. This may be due to surface lone pairs of electrons, which can be confirmed by the specific effective charge number of the M atoms of the M_2_C-type (Figure 8b). The novel composite Ti_3_C_2_/PEI/BO was prepared by two-step in situ polymerization with excellent wear resistance and long-term cycling stability and has an adsorption capacity of 2.60 mmol g^−1^ for CO_2_. The abundant -OH groups on the surface of MXene can directly initiate the polymerization of aziridines, and PEI is covalently bonded to the surface of MXene by a strong chemical bond between the organic amine and the support. The low regeneration heat and stability of the material make it an excellent adsorbent for CO_2_ capture [133].

For the conversion of CO_2_ into a valuable resource, MXene (Pd_50_-Ru_50_/MXene) nanocatalysts were microwave synthesized for the selective production of methanol by CO_2_ hydrogenation. On the Pd_50_-Ru_50_/MXene catalyst, the yield of CO conversion to CH_3_OH was about 76%, and H_2_ was produced in situ at 120 °C for up to 12 h, with a final CO_2_ conversion rate of 78%. The Pd_50_-Ru_50_/MXene catalysts showed good catalytic stability after repeated use [134]. Combining electrochemical CO_2_ conversion provides power while producing valuable chemicals. The parallel-arranged tubular structure structured by two-dimensional Ti_3_C_2_T_x_ MXene/carbon heterostructure provides a potential of 1.38 V at 0.2 A·g^−1^ [135]. Theoretical calculations prove that the high stability and catalytic activity of Ti_3_C_2_T_x_ MXene is beneficial to carbon dioxide capture. Single-atom catalysts exhibit exceptional electrocatalytic activity. SA-Cu-MXene materials were prepared for electrocatalytic CO_2_ reduction to methanol. The hybrid A layer (Al and Cu) in the quaternary MAX phase (Ti_3_(Al_1 − x_Cu_x_)C_2_) was selectively etched, and the Cu atoms were retained and immobilized after removing the Al atoms. The resulting single-atom Cu catalyst demonstrated an efficiency of 59.1% in producing methanol and exhibited excellent catalytic stability [136].

**Figure 9:**
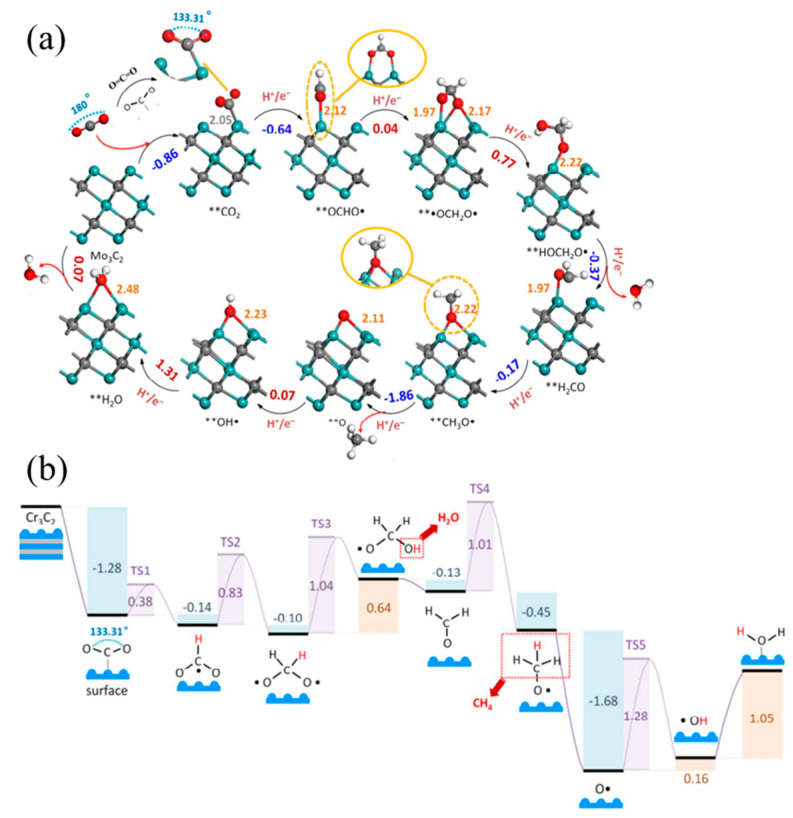
(**a**) The side view of minimum energy path (PBE/DFT-D3 calculations) followed for the CO_2_ conversion mechanism into *CH_4_ and **H_2_O catalyzed by Mo_3_C_2_. Note: Gray, lilac, red, and white spheres refer to C, Mo, O, and H atoms, respectively; ** refers to chemisorbed species. Selected distances are indicated in Å. The gray number is Mo–C bond length, while the orange numbers are Mo–O bond lengths; the blue numbers are energy spontaneously released by reactions, while the red numbers represent the energy required to carry out a reaction step (in eV). (**b**) Minimum energy path for the CO_2_ conversion into *CH_4_ and **H_2_O catalyzed by Cr_3_C_2_ calculated with PBE/DFT-D3 [131].

Regarding theoretical mechanism calculations, Li et al. [131] predicted that two-dimensional transition metal carbides of group IV, V, and VI (MXene) with the molecular formula M_3_C_2_ can catalyze the conversion of CO_2_ to hydrocarbons and selectively form CH_4_ (Figure 9a). The most promising choices are MXene Cr_3_C_2_ and Mo_3_C_2_. When the surfaces of MXene are capped with -O or -OH, the energy cost can be further reduced to 0.35 eV and 0.54 eV. The reactivity of the material is improved by doping it with O. In general, the first hydrogenation of CO_2_ to form the OCHO- radical requires a large energy input in the isolated conversion. However, for MXene, a spontaneous reaction energy of −0.14 eV was observed for Cr_3_C_2_. The structures and energies of the transition states (TS) were determined by DFT-D3 calculations. The activation barriers for TS1 and TS2 are only 0.38 and 0.83 eV. The electrochemistry of the reduction of **•OCH_2_O• to **HOCH_2_O• in TS3 is presented. It was found that this reduction has an increased energy barrier of 1.04 eV. Subsequently, H_2_O is spontaneously released from **HOCH_2_O• to produce **H_2_CO, without requiring an energy barrier. The electrochemical reduction of **H_2_CO to **CH_3_O• in TS4 has a significant potential barrier of 1.01 eV and exhibits similar properties to TS3 [131] (Figure 9b).

**Table 5:** Summary of typical examples of MXenes-based carbon capture catalysts.

Catalysts	MXenes	Catalytic Activity	Ref.
AC-MX-x	Ti_2_CT_x_	At 2.5 wt.% MXene, the adsorption capacity of AC increased from 46.46 cm^3^/g to 67.83 cm^3^/g.	[137]
AC/MXene sandwich	Ti_3_C_2_T_x_	MXene containing ~4% has significant CO_2_ adsorption capacity (~8.9 mg/g).	[138]
Pebax/CMC@MXene MMMs	Ti_3_C_2_T_x_	The CO_2_/N_2_ adsorption selectivity is 40.1, and after 60 h of testing, its separation performance has no significant change.	[139]
MXene-FO membrane	Ti_3_C_2_T_x_	MXene sandwich TFC-FO membrane achieves higher water flux and lower specific solute flux.	[140]
Cr_3_C_2_	Cr_3_C_2_	The reaction energy value of Cr_3_C_2_ MXene is 1.05 eV, and the overpotential when the maximum Faradaic efficiency of CO_2_ to CO acts is 540 mV.	[131]
Pd-MXene	Pd-MXene	Displays a specific surface area of 97.5 m^2^g^−1^ and multiple pores and selectively electroreduces CO_2_ to CH_3_OH via multiple electron transfer.	[141]
Pd_50_-Ru_50_/MXene	Ti_3_C_2_T_x_	The CO_2_ conversion efficiency of the Pd_50_-Ru_50_/MXene catalyst is as high as 78%, and the CH_3_OH yield is 76%.	[134]
MXene@CNF-3 membrane	Ti_3_C_2_T_x_	The CO_2_ permeability is 156.7 Barrer, and the CO_2_/N_2_ and CO_2_/CH_4_ selectivities are 42.6 and 47.8, respectively.	[142]
MX-fluid-M2070	Ti_3_C_2_T_x_	Compared with pure epoxy resin, the flexural strength, flexural modulus, and impact strength increased by 15.32%, 6.42%, and 110.31%, respectively.	[143]
Pebax-Ti_3_C_2_T_x_TFC membranes	Ti_3_C_2_T_x_	The composite membrane exhibits efficient CO_2_ permeability (1986.5 GPU) and CO_2_/N_2_ selectivity ≈ 42.	[144]
T-SDESM	Ti_3_C_2_T_x_	The permeability of CO_2_ is approximately 26.35 GPU, and the selectivities for N_2_, CH_4_, and H_2_ are 319.15, 249.01, and 12.38, respectively.	[145]
MXene/PEG (600)	Ti_3_C_2_T_x_	The mixed matrix membrane exhibits a CO_2_ permeability of 1626.99 GPU and CO_2_/N_2_ and CO_2_/CH_4_ selectivities of 32.18 and 27.84, respectively.	[146]

## 4. Conclusions and Prospect

This paper mainly reviews the current structural features, synthetic strategies of MXenes, and their applications and advances in catalysis. First, the structural features and morphological characteristics, corresponding elemental combinations of MXene are summarized. The synthetic methods of MXenes, including acid etching, fluoride etching, and electrochemical etching, are summarized. In particular, different etching environments and etchant concentrations produce different morphologies and tunable surface chemistry of layered MXenes. Depending on the research topic, appropriate etching methods can be used. In terms of catalysis applications, the use of MXenes in electrochemical reduction, photocatalysis, energy conversion and storage, and carbon capture and conversion are highlighted and discussed. Recent research advances in composite functional catalytic materials combined with MXene are systematically presented. The production of catalytically valuable compounds, composite energy storage and conversion are introduced, and relevant computational models for predicting and simulating catalytic performance are also presented.

MXenes are a type of 2D nanomaterials that have demonstrated superior optical, electrical, and physical properties. They have made significant contributions to various fields over the past decade. The number of related publications has been increasing year by year. MXene-based materials offer promising opportunities for the development of energy storage and conversion, pollutant degradation, and environmental protection. However, as the research on MXenes is still in the exploratory stage, there remain numerous challenges and bottlenecks that have yet to be resolved. MXene itself also exhibits several shortcomings, such as susceptibility to accumulation and structural instability in oxygen-rich environments, which restricts its large-scale application. In summary, further research and practical applications are needed to develop commercially available multifunctional MXenes that can outperform traditional materials at a competitive price. The current challenges faced by MXene research and the directions that require in-depth research in the future are as follows:

(1) To promote the industrial use of MXene in the future, it is necessary to develop tunable, low-cost, safe, and environmentally friendly synthesis processes. Additionally, composite materials with improved electrical, magnetic, and optical effects should be developed.

(2) It is important to systematically study the environmental toxicity and life cycle assessment of these materials to ensure their environmental safety and compatibility with the ecosystem.

(3) Explore the potential of MXene in expanding its application fields and enhancing material properties through special structural modification and multi-functional composites. Conduct exploratory applications in the fields of healthcare, seawater desalination, functional sensors, and satellite communications, while considering the actual application of the industry.

(4) To improve the subsequent catalytic applications, it is necessary to gain a deeper understanding of the internal mechanisms of catalysis and degradation, as well as the micro-mechanisms within functional MXene-based composites. This includes understanding the active sites of MXene catalysis and their regulation. By deepening our understanding, we can better use theory to guide the direction of catalytic applications.

(5) To improve the performance of 2D MXenes, advanced characterization methods and computational simulation models can be developed and used to explore the internal evolution of the catalytic process of MXene composite functional materials from a microscopic and modeling perspective. This will allow for a more accurate quantification of the course of the chemical reaction.

Since its discovery in 2011, MXene has been extensively researched and applied in the field of functional materials due to its unique electro-optical properties, tunability, and combinatorial position diversity. MXene-based materials are widely used in electrocatalysis, photocatalysis, and energy storage as catalysts or cocatalysts. The excellent properties of MXene-based materials show great potential to replace traditional precious metals. Academia and industry are expected to collaborate to expand and deepen MXene research in the field of catalysis, leading to its industrial application.

## Figures and Tables

**Table 2:** Summary of typical examples of MXenes-based composite electrocatalysts.

Electrocatalysts	MXene	Catalytic Activity	Ref.
Co_2_P/N@Ti_3_C_2_T_x_@NF	Ti_3_C_2_T_x_	Ultra-low overpotential of 15 mV, achieving a current density of 10 mA∙cm^−2^.	[67]
Pt@Ti_3_C_2_Tx	Ti_3_C_2_T_x_	1% Pt@MXene can completely reduce CAP by 98.7% within 90 min and maintain 86.5% after 25 cycles.	[68]
Ti_3_C_2_@mNiCoP	Ti_3_C_2_	Water splitting performance remains unchanged after 12 h of operation.	[69]
Pd-MXene	Ti_3_C_2_T_x_	Excellent nitrate yield (2.80 µg h^−1^ mg_cat_ ^−1^) and Faradaic efficiency (11.34%).	[70]
NiFeP/MXene	Ti_3_C_2_	Exhibiting a low overpotential of 286 mV at 10 mA∙cm^−2^ and a current density of 10 mA∙cm^−2^ at a cell voltage of 1.61 V.	[71]
CdS/Ti_3_C_2_	Ti_3_C_2_	Faradaic efficiency is as high as 94% at −1.0 V.	[72]
Mo_2_TiC_2_	Mo_2_TiC_2_	The overpotential is 0.26 V with high NRR activity, which meets the balance of N_2_ activation and overpotential reduction.	[73]
MXene/NW-Ag_0.9_Ti_0.1_	Ti_3_C_2_	It exhibits onset potential (EORR) and half-wave potential (E1/2) at 1600 rpm, which are 0.921 V (RHE) and 0.782 V (RHE), respectively.	[74]
Mo_2_CT_x_/2H-MoS_2_	Mo_2_CT_x_	Maintains current densities in excess of −450 mA∙cm^−2^_geom_ with less than 30 mV overpotential decay after 100,000 consecutive cyclic voltammetry cycles.	[75]
NiS_2_/V-MXene	Ti_3_C_2_T_x_	The overpotential is 179 mV to achieve a catalytic current density of −10 mA∙cm^2^.	[76]
FeNi-LDH/Ti_3_C_2_-MXene	Ti_3_C_2_	A current density of 50 mA∙cm^−2^ is achieved at η = 370 mV.	[77]

## Data Availability

Not applicable.

## References

[1] Raffa P. (2021). Where is research on fossil fuels going in times of climate change? A perspective on chemical enhanced oil recovery. MRS Commun..

[2] Chandrasekar K., Sudhakar S., Rajappan R., Senthil S., Balu P. (2022). Present developments and the reach of alternative fuel: A review. Mater. Today Proc..

[3] Naguib M., Kurtoglu M., Presser V., Lu J., Niu J., Heon M., Hultman L., Gogotsi Y., Barsoum M.W. (2011). Two-dimensional nanocrystals produced by exfoliation of Ti_3_AlC_2_. Adv. Mater..

[4] Ronchi R.M., Arantes J.T., Santos S.F. (2019). Synthesis, structure, properties and applications of MXenes: Current status and perspectives. Ceram. Int..

[5] Salim O., Mahmoud K., Pant K., Joshi R. (2019). Introduction to MXenes: Synthesis and characteristics. Mater. Today Chem..

[6] Nan J., Guo X., Xiao J., Li X., Chen W., Wu W., Liu H., Wang Y., Wu M., Wang G. (2021). Nanoengineering of 2D MXene-based materials for energy storage applications. Small.

[7] Shuai T.-Y., Qi-Ni Z., Xu H., Huang C.-J., Zhi-Jie Z., Li G.-R. (2023). Recent Advances in the Synthesis and Electrocatalytic Applications of MXene Materials. Chem. Commun..

[8] Zhong Q., Li Y., Zhang G. (2021). Two-dimensional MXene-based and MXene-derived photocatalysts: Recent developments and perspectives. Chem. Eng. J..

[9] Hui X., Ge X., Zhao R., Li Z., Yin L. (2020). Interface chemistry on MXene-based materials for enhanced energy storage and conversion performance. Adv. Funct. Mater..

[10] Sharif H.M.A., Rashad M., Hussain I., Abbas A., Aldosari O.F., Li C. (2023). Green energy Harvesting from CO_2_ and NO_x_ by MXene materials: Detailed Historical and Future Prospective. Appl. Catal. B Environ..

[11] Wang H., Lee J.-M. (2020). Recent advances in structural engineering of MXene electrocatalysts. J. Mater. Chem. A.

[12] Kuang P., Low J., Cheng B., Yu J., Fan J. (2020). MXene-based photocatalysts. J. Mater. Sci. Technol..

[13] Li K., Liang M., Wang H., Wang X., Huang Y., Coelho J., Pinilla S., Zhang Y., Qi F., Nicolosi V. (2020). 3D MXene architectures for efficient energy storage and conversion. Adv. Funct. Mater..

[14] Chen Y., Liu C., Guo S., Mu T., Wei L., Lu Y. (2022). CO_2_ capture and conversion to value-added products promoted by MXene-based materials. Green Energy Environ..

[15] Devaraj M., Rajendran S., Hoang T.K., Soto-Moscoso M. (2022). A review on MXene and its nanocomposites for the detection of toxic inorganic gases. Chemosphere.

[16] Malaki M., Varma R.S. (2020). Mechanotribological aspects of MXene-reinforced nanocomposites. Adv. Mater..

[17] Biswal L., Mohanty R., Nayak S., Parida K. (2022). Review on MXene/TiO_2_ nanohybrids for photocatalytic hydrogen production and pollutant degradations. J. Environ. Chem. Eng..

[18] Ding B., Ong W.-J., Jiang J., Chen X., Li N. (2020). Uncovering the electrochemical mechanisms for hydrogen evolution reaction of heteroatom doped M_2_C MXene (M = Ti, Mo). Appl. Surf. Sci..

[19] Pu L., Zhang J., Jiresse N.K.L., Gao Y., Zhou H., Naik N., Gao P., Guo Z. (2022). N-doped MXene derived from chitosan for the highly effective electrochemical properties as supercapacitor. Adv. Compos. Hybrid Mater..

[20] Hong X., Lu Z., Zhao Y., Lyu L., Ding L., Wei Y., Wang H. (2022). Fast fabrication of freestanding MXene-ZIF-8 dual-layered membranes for H_2_/CO_2_ separation. J. Membr. Sci..

[21] Fan W.K., Sherryna A., Tahir M. (2022). Advances in Titanium Carbide (Ti_3_C_2_T_x_) MXenes and Their Metal–Organic Framework (MOF)-Based Nanotextures for Solar Energy Applications: A Review. ACS Omega.

[22] Kang Z., Khan M.A., Gong Y., Javed R., Xu Y., Ye D., Zhao H., Zhang J. (2021). Recent progress of MXenes and MXene-based nanomaterials for the electrocatalytic hydrogen evolution reaction. J. Mater. Chem. A.

[23] Johnson L.R., Sridhar S., Zhang L., Fredrickson K.D., Raman A.S., Jang J., Leach C., Padmanabhan A., Price C.C., Frey N.C. (2019). MXene materials for the electrochemical nitrogen reduction—Functionalized or not?. ACS Catal..

[24] Li X., Huang Z., Shuck C.E., Liang G., Gogotsi Y., Zhi C. (2022). MXene chemistry, electrochemistry and energy storage applications. Nat. Rev. Chem..

[25] Zubair M., Hassan M.M.U., Mehran M.T., Baig M.M., Hussain S., Shahzad F. (2022). 2D MXenes and their heterostructures for HER, OER and overall water splitting: A review. Int. J. Hydrogen Energy.

[26] Zhan X., Si C., Zhou J., Sun Z. (2020). MXene and MXene-based composites: Synthesis, properties and environment-related applications. Nanoscale Horiz..

[27] Lei D., Liu N., Su T., Zhang Q., Wang L., Ren Z., Gao Y. (2022). Roles of MXene in pressure sensing: Preparation, composite structure design, and mechanism. Adv. Mater..

[28] Naguib M., Barsoum M.W., Gogotsi Y. (2021). Ten years of progress in the synthesis and development of MXenes. Adv. Mater..

[29] Gogotsi Y., Anasori B. (2019). The Rise of MXenes.

[30] Wei Y., Zhang P., Soomro R.A., Zhu Q., Xu B. (2021). Advances in the synthesis of 2D MXenes. Adv. Mater..

[31] Bharali L., Kalita J., Sankar Dhar S. (2023). Several fundamental aspects of MXene: Synthesis and their applications. ChemistrySelect.

[32] Murali G., Reddy Modigunta J.K., Park Y.H., Lee J.-H., Rawal J., Lee S.-Y., In I., Park S.-J. (2022). A review on MXene synthesis, stability, and photocatalytic applications. ACS Nano.

[33] Alhabeb M., Maleski K., Anasori B., Lelyukh P., Clark L., Sin S., Gogotsi Y. (2017). Guidelines for synthesis and processing of two-dimensional titanium carbide (Ti_3_C_2_T_x_ MXene). Chem. Mater..

[34] Anasori B., Xie Y., Beidaghi M., Lu J., Hosler B.C., Hultman L., Kent P.R., Gogotsi Y., Barsoum M.W. (2015). Two-dimensional, ordered, double transition metals carbides (MXenes). ACS Nano.

[35] Anayee M., Kurra N., Alhabeb M., Seredych M., Hedhili M.N., Emwas A.-H., Alshareef H.N., Anasori B., Gogotsi Y. (2020). Role of acid mixtures etching on the surface chemistry and sodium ion storage in Ti_3_C_2_T_x_ MXene. Chem. Commun..

[36] Wang C., Shou H., Chen S., Wei S., Lin Y., Zhang P., Liu Z., Zhu K., Guo X., Wu X. (2021). HCl-Based hydrothermal etching strategy toward fluoride-free MXenes. Adv. Mater..

[37] Liu F., Zhou A., Chen J., Jia J., Zhou W., Wang L., Hu Q. (2017). Preparation of Ti_3_C_2_ and Ti_2_C MXenes by fluoride salts etching and methane adsorptive properties. Appl. Surf. Sci..

[38] Liu L., Zschiesche H., Antonietti M., Gibilaro M., Chamelot P., Massot L., Rozier P., Taberna P.L., Simon P. (2023). In situ synthesis of MXene with tunable morphology by electrochemical etching of MAX phase prepared in molten salt. Adv. Energy Mater..

[39] Yin T., Li Y., Wang R., Al-Hartomy O.A., Al-Ghamdi A., Wageh S., Luo X., Tang X., Zhang H. (2021). Synthesis of Ti3C2Fx MXene with controllable fluorination by electrochemical etching for lithium-ion batteries applications. Ceram. Int..

[40] Lim K.R.G., Shekhirev M., Wyatt B.C., Anasori B., Gogotsi Y., Seh Z.W. (2022). Fundamentals of MXene synthesis. Nat. Synth..

[41] Kumar S. (2023). Fluorine-Free MXenes: Recent Advances, Synthesis Strategies, and Mechanisms. Small.

[42] Jiang Q., Lei Y., Liang H., Xi K., Xia C., Alshareef H.N. (2020). Review of MXene electrochemical microsupercapacitors. Energy Storage Mater..

[43] Zhang N., Hong Y., Yazdanparast S., Zaeem M.A. (2018). Superior structural, elastic and electronic properties of 2D titanium nitride MXenes over carbide MXenes: A comprehensive first principles study. 2D Mater..

[44] Hassan N., Jalil A., Rajendran S., Khusnun N., Bahari M., Johari A., Kamaruddin M., Ismail M. (2023). Recent review and evaluation of green hydrogen production via water electrolysis for a sustainable and clean energy society. Int. J. Hydrogen Energy.

[45] Burton N., Padilla R., Rose A., Habibullah H. (2021). Increasing the efficiency of hydrogen production from solar powered water electrolysis. Renew. Sustain. Energy Rev..

[46] Chi J., Yu H. (2018). Water electrolysis based on renewable energy for hydrogen production. Chin. J. Catal..

[47] Mohammed-Ibrahim J., Sun X. (2019). Recent progress on earth abundant electrocatalysts for hydrogen evolution reaction (HER) in alkaline medium to achieve efficient water splitting—A review. J. Energy Chem..

[48] Verma J., Goel S. (2022). Cost-effective electrocatalysts for hydrogen evolution reactions (HER): Challenges and prospects. Int. J. Hydrogen Energy.

[49] Cui C., Cheng R., Zhang H., Zhang C., Ma Y., Shi C., Fan B., Wang H., Wang X. (2020). Ultrastable MXene@Pt/SWCNTs’ nanocatalysts for hydrogen evolution reaction. Adv. Funct. Mater..

[50] Lv Z., Wang M., Liu D., Jian K., Zhang R., Dang J. (2021). Synergetic effect of Ni_2_P and MXene enhances catalytic activity in the hydrogen evolution reaction. Inorg. Chem..

[51] Tang Y., Yang C., Sheng M., Yin X., Que W. (2020). Synergistically Coupling phosphorus-doped molybdenum carbide with Mxene as a highly efficient and stable electrocatalyst for hydrogen evolution reaction. ACS Sustain. Chem. Eng..

[52] Gan J., Li F., Tang Q. (2021). Vacancies-Engineered M_2_CO_2_ MXene as an efficient hydrogen evolution reaction electrocatalyst. J. Phys. Chem. Lett..

[53] Arif M., Babar M., Azhar U., Sagir M., Tahir M.B., Mushtaq M.A., Yasin G., Mubashir M., Chong J.W.R., Khoo K.S. (2023). Rational design and modulation strategies of Mo-based electrocatalysts and photo/electrocatalysts towards nitrogen reduction to ammonia (NH_3_). Chem. Eng. J..

[54] Islam J., Shareef M., Zabed H.M., Qi X., Chowdhury F.I., Das J., Uddin J., Kaneti Y.V., Khandaker M.U., Ullah M.H. (2023). Electrochemical nitrogen fixation in metal-N_2_ batteries: A paradigm for simultaneous NH_3_ synthesis and energy generation. Energy Storage Mater..

[55] Lv X.W., Weng C.C., Yuan Z.Y. (2020). Ambient ammonia electrosynthesis: Current status, challenges, and perspectives. ChemSusChem.

[56] Estevez R., López-Tenllado F.J., Aguado-Deblas L., Bautista F.M., Romero A.A., Luna D. (2023). Current research on green ammonia (nh3) as a potential vector energy for power storage and engine fuels: A review. Energies.

[57] Sonker M., Tiwary S.K., Shreyash N., Bajpai S., Ray M., Kar S.K., Balathanigaimani M. (2022). Ammonia as an alternative fuel for vehicular applications: Paving the way for adsorbed ammonia and direct ammonia fuel cells. J. Clean. Prod..

[58] Amrillah T., Hermawan A., Alviani V.N., Seh Z.W., Yin S. (2021). MXenes and their derivatives as nitrogen reduction reaction catalysts: Recent progress and perspectives. Mater. Today Energy.

[59] Kui P., Liming H., Jiada H., Guanhua Z., Leiming T. (2024). Highly efficient electrochemical reduction of nitrate to ammonia on cobalt doped Ti_3_C_2_ MXene nanosheets. Inorg. Chem. Commun..

[60] Johnson D., Hunter B., Christie J., King C., Kelley E., Djire A. (2022). Ti_2_N nitride MXene evokes the Mars-van Krevelen mechanism to achieve high selectivity for nitrogen reduction reaction. Sci. Rep..

[61] Peng W., Luo M., Xu X., Jiang K., Peng M., Chen D., Chan T.S., Tan Y. (2020). Spontaneous atomic ruthenium doping in Mo_2_CT_X_ MXene defects enhances electrocatalytic activity for the nitrogen reduction reaction. Adv. Energy Mater..

[62] Fang Q., Gao Y., Zhang W., Sun F., Pan J., Zhuang G., Deng S., Yao Z., Wang J. (2021). Oxygen groups enhancing the mechanism of nitrogen reduction reaction properties on Ru-or Fe-supported Nb_2_C MXene. J. Phys. Chem. C.

[63] Guo Y., Wang T., Yang Q., Li X., Li H., Wang Y., Jiao T., Huang Z., Dong B., Zhang W. (2020). Highly efficient electrochemical reduction of nitrogen to ammonia on surface termination modified Ti_3_C_2_T_x_ MXene nanosheets. ACS Nano.

[64] Shi Y., Liu Y. (2021). Vacancy and N dopants facilitated Ti^3+^ sites activity in 3D Ti_3 − x_C_2_T_y_ MXene for electrochemical nitrogen fixation. Appl. Catal. B Environ..

[65] Chu K., Li X., Li Q., Guo Y., Zhang H. (2021). Synergistic enhancement of electrocatalytic nitrogen reduction over boron nitride quantum dots decorated Nb_2_CT_x_-MXene. Small.

[66] Jin Z., Liu C., Liu Z., Han J., Fang Y., Han Y., Niu Y., Wu Y., Sun C., Xu Y. (2020). Rational design of hydroxyl-rich Ti_3_C_2_T_x_ MXene quantum dots for high-performance electrochemical N_2_ reduction. Adv. Energy Mater..

[67] Lv Z., Ma W., Wang M., Dang J., Jian K., Liu D., Huang D. (2021). Co-constructing interfaces of multiheterostructure on MXene (Ti_3_C_2_T_x_)-modified 3D self-supporting electrode for ultraefficient electrocatalytic HER in alkaline media. Adv. Funct. Mater..

[68] Li L.-X., Zhang G.-C., Sun W.-J., Zhang H.-Y., Wang S.-X., Wei J.-L., He J.-H., Song K., Lu J.-M. (2022). Construction of ultra-small Pt nanoparticles@ Ti_3_C_2_T_x_ MXene electrocatalyst for efficient and stable electrochemical hydrodechlorination of chloramphenicol. Chem. Eng. J..

[69] Yue Q., Sun J., Chen S., Zhou Y., Li H., Chen Y., Zhang R., Wei G., Kang Y. (2020). Hierarchical mesoporous MXene–NiCoP electrocatalyst for water-splitting. ACS Appl. Mater. Interfaces.

[70] Fang W., Du C., Kuang M., Chen M., Huang W., Ren H., Xu J., Feldhoff A., Yan Q. (2020). Boosting efficient ambient nitrogen oxidation by a well-dispersed Pd on MXene electrocatalyst. Chem. Commun..

[71] Chen J., Long Q., Xiao K., Ouyang T., Li N., Ye S., Liu Z.-Q. (2021). Vertically-interlaced NiFeP/MXene electrocatalyst with tunable electronic structure for high-efficiency oxygen evolution reaction. Sci. Bull..

[72] Wang Y., Du R., Li Z., Song H., Chao Z., Zu D., Chong D., Gao N., Li C. (2021). Rationally designed CdS/Ti_3_C_2_ MXene electrocatalysts for efficient CO2 reduction in aqueous electrolyte. Ceram. Int..

[73] Gao Y., Cao Y., Zhuo H., Sun X., Gu Y., Zhuang G., Deng S., Zhong X., Wei Z., Li X. (2020). Mo2TiC2 MXene: A promising catalyst for electrocatalytic ammonia synthesis. Catal. Today.

[74] Zhang Z., Li H., Zou G., Fernandez C., Liu B., Zhang Q., Hu J., Peng Q. (2016). Self-reduction synthesis of new MXene/Ag composites with unexpected electrocatalytic activity. ACS Sustain. Chem. Eng..

[75] Lim K.R.G., Handoko A.D., Johnson L.R., Meng X., Lin M., Subramanian G.S., Anasori B., Gogotsi Y., Vojvodic A., Seh Z.W. (2020). 2h-Mos2 on Mo2ct X Mxene nanohybrid for efficient and durable electrocatalytic hydrogen evolution. ACS Nano.

[76] Kuang P., He M., Zhu B., Yu J., Fan K., Jaroniec M. (2019). 0D/2D NiS_2_/V-MXene composite for electrocatalytic H_2_ evolution. J. Catal..

[77] Yu M., Zhou S., Wang Z., Zhao J., Qiu J. (2018). Boosting electrocatalytic oxygen evolution by synergistically coupling layered double hydroxide with MXene. Nano Energy.

[78] Guo Q., Zhou C., Ma Z., Yang X. (2019). Fundamentals of TiO_2_ photocatalysis: Concepts, mechanisms, and challenges. Adv. Mater..

[79] Meng A., Zhang L., Cheng B., Yu J. (2019). Dual cocatalysts in TiO_2_ photocatalysis. Adv. Mater..

[80] Naldoni A., Altomare M., Zoppellaro G., Liu N., Kment S., Zboril R., Schmuki P. (2018). Photocatalysis with reduced TiO_2_: From black TiO_2_ to cocatalyst-free hydrogen production. ACS Catal..

[81] Ma D., Zhai S., Wang Y., Liu A., Chen C. (2019). TiO_2_ photocatalysis for transfer hydrogenation. Molecules.

[82] Rengifo-Herrera J.A., Pulgarin C. (2023). Why five decades of massive research on heterogeneous photocatalysis, especially on TiO_2_, has not yet driven to water disinfection and detoxification applications? Critical review of drawbacks and challenges. Chem. Eng. J..

[83] Peiris S., de Silva H.B., Ranasinghe K.N., Bandara S.V., Perera I.R. (2021). Recent development and future prospects of TiO_2_ photocatalysis. J. Chin. Chem. Soc..

[84] Xie X., Zhang N. (2020). Positioning MXenes in the photocatalysis landscape: Competitiveness, challenges, and future perspectives. Adv. Funct. Mater..

[85] Kim H., Anasori B., Gogotsi Y., Alshareef H.N. (2017). Thermoelectric properties of two-dimensional molybdenum-based MXenes. Chem. Mater..

[86] Low J., Zhang L., Tong T., Shen B., Yu J. (2018). TiO_2_/MXene Ti_3_C_2_ composite with excellent photocatalytic CO_2_ reduction activity. J. Catal..

[87] Han X., An L., Hu Y., Li Y., Hou C., Wang H., Zhang Q. (2020). Ti_3_C_2_ MXene-derived carbon-doped TiO_2_ coupled with g-C_3_N_4_ as the visible-light photocatalysts for photocatalytic H_2_ generation. Appl. Catal. B Environ..

[88] Wu Z., Liang Y., Yuan X., Zou D., Fang J., Jiang L., Yang H., Xiao Z. (2020). MXene Ti_3_C_2_ derived Z–scheme photocatalyst of graphene layers anchored TiO_2_/g–C_3_N_4_ for visible light photocatalytic degradation of refractory organic pollutants. Chem. Eng. J..

[89] Sergiienko S.A., Tobaldi D.M., Lajaunie L., Lopes D.V., Constantinescu G., Shaula A.L., Shcherban N.D., Shkepu V.I., Calvino J.J., Frade J.R. (2022). Photocatalytic removal of benzene over Ti_3_C_2_T_x_ MXene and TiO_2_–MXene composite materials under solar and NIR irradiation. J. Mater. Chem. C.

[90] Liu X., Chen C. (2020). Mxene enhanced the photocatalytic activity of ZnO nanorods under visible light. Mater. Lett..

[91] Nasri M.S.I., Samsudin M.F.R., Tahir A.A., Sufian S. (2022). Effect of MXene loaded on g-C_3_N_4_ photocatalyst for the photocatalytic degradation of methylene blue. Energies.

[92] Othman Z., Sinopoli A., Mackey H.R., Mahmoud K.A. (2021). Efficient Photocatalytic Degradation of Organic Dyes by AgNPs/TiO_2_/Ti_3_C_2_T_x_ MXene Composites under UV and Solar Light. ACS Omega.

[93] Zuo G., Wang Y., Teo W.L., Xie A., Guo Y., Dai Y., Zhou W., Jana D., Xian Q., Dong W. (2020). Ultrathin ZnIn_2_S_4_ nanosheets anchored on Ti_3_C_2_T_X_ MXene for photocatalytic H_2_ evolution. Angew. Chem..

[94] Jin S., Jing H., Wang L., Hu Q., Zhou A. (2022). Construction and performance of CdS/MoO_2_@Mo_2_C-MXene photocatalyst for H2 production. J. Adv. Ceram..

[95] Peng C., Xie X., Xu W., Zhou T., Wei P., Jia J., Zhang K., Cao Y., Wang H., Peng F. (2021). Engineering highly active Ag/Nb_2_O_5_@Nb_2_CT_x_ (MXene) photocatalysts via steering charge kinetics strategy. Chem. Eng. J..

[96] Li Y., Yin Z., Ji G., Liang Z., Xue Y., Guo Y., Tian J., Wang X., Cui H. (2019). 2D/2D/2D heterojunction of Ti_3_C_2_ MXene/MoS_2_ nanosheets/TiO_2_ nanosheets with exposed (001) facets toward enhanced photocatalytic hydrogen production activity. Appl. Catal. B Environ..

[97] Cheng L., Chen Q., Li J., Liu H. (2020). Boosting the photocatalytic activity of CdLa_2_S_4_ for hydrogen production using Ti_3_C_2_ MXene as a co-catalyst. Appl. Catal. B Environ..

[98] Cai T., Wang L., Liu Y., Zhang S., Dong W., Chen H., Yi X., Yuan J., Xia X., Liu C. (2018). Ag_3_PO_4_/Ti_3_C_2_ MXene interface materials as a Schottky catalyst with enhanced photocatalytic activities and anti-photocorrosion performance. Appl. Catal. B Environ..

[99] Li H., Sun B., Gao T., Li H., Ren Y., Zhou G. (2022). Ti_3_C_2_ MXene co-catalyst assembled with mesoporous TiO_2_ for boosting photocatalytic activity of methyl orange degradation and hydrogen production. Chin. J. Catal..

[100] Ran J., Gao G., Li F.-T., Ma T.-Y., Du A., Qiao S.-Z. (2017). Ti3C2 MXene co-catalyst on metal sulfide photo-absorbers for enhanced visible-light photocatalytic hydrogen production. Nat. Commun..

[101] Li Y., Ding L., Liang Z., Xue Y., Cui H., Tian J. (2020). Synergetic effect of defects rich MoS_2_ and Ti_3_C_2_ MXene as cocatalysts for enhanced photocatalytic H_2_ production activity of TiO_2_. Chem. Eng. J..

[102] Wojciechowski T., Rozmysłowska-Wojciechowska A., Matyszczak G., Wrzecionek M., Olszyna A., Peter A., Mihaly-Cozmuta A., Nicula C., Mihaly-Cozmuta L., Podsiadło S. (2019). Ti_2_C MXene modified with ceramic oxide and noble metal nanoparticles: Synthesis, morphostructural properties, and high photocatalytic activity. Inorg. Chem..

[103] Cui C., Guo R., Xiao H., Ren E., Song Q., Xiang C., Lai X., Lan J., Jiang S. (2020). Bi_2_WO_6_/Nb_2_CT_x_ MXene hybrid nanosheets with enhanced visible-light-driven photocatalytic activity for organic pollutants degradation. Appl. Surf. Sci..

[104] Guo S., Luo H., Bao Y., Li Y., Guan H., Zhu Y. (2022). Construction of hierarchical Ti_3_C_2_T_x_ MXene/ZnIn_2_S_4_ heterostructures for efficiently photocatalytic reduction of Cr (VI) under visible light. Appl. Surf. Sci..

[105] Zhang N., Chen X., Yu M., Niu Z., Cheng F., Chen J. (2020). Materials chemistry for rechargeable zinc-ion batteries. Chem. Soc. Rev..

[106] Das P., Wu Z.-S. (2020). MXene for energy storage: Present status and future perspectives. J. Phys. Energy.

[107] Wei C., Xi B., Wang P., Wang Z., An X., Tian K., Feng J., Xiong S. (2023). In Situ Growth Engineering on 2D MXenes for Next-Generation Rechargeable Batteries. Adv. Energy Sustain. Res..

[108] Liao L., Jiang D., Zheng K., Zhang M., Liu J. (2021). Industry-scale and environmentally stable Ti_3_C_2_T_x_ MXene based film for flexible energy storage devices. Adv. Funct. Mater..

[109] Lin P., Xie J., He Y., Lu X., Li W., Fang J., Yan S., Zhang L., Sheng X., Chen Y. (2020). MXene aerogel-based phase change materials toward solar energy conversion. Sol. Energy Mater. Sol. Cells.

[110] Gong S., Ding Y., Li X., Liu S., Wu H., Lu X., Qu J. (2021). Novel flexible polyurethane/MXene composites with sensitive solar thermal energy storage behavior. Compos. Part A.

[111] Luo Y., Xie Y., Jiang H., Chen Y., Zhang L., Sheng X., Xie D., Wu H., Mei Y. (2021). Flame-retardant and form-stable phase change composites based on MXene with high thermostability and thermal conductivity for thermal energy storage. Chem. Eng. J..

[112] Zheng S., Wang H., Das P., Zhang Y., Cao Y., Ma J., Liu S., Wu Z.S. (2021). Multitasking MXene inks enable high-performance printable microelectrochemical energy storage devices for all-flexible self-powered integrated systems. Adv. Mater..

[113] Qian A., Pang Y., Wang G., Hao Y., Liu Y., Shi H., Chung C.-H., Du Z., Cheng F. (2020). Pseudocapacitive charge storage in MXene–V_2_O_5_ for asymmetric flexible energy storage devices. ACS Appl. Mater. Interfaces.

[114] Zhu X., Huang X., Zhao R., Liao K., Chan V. (2020). Dual-functional Ti_3_C_2_T_x_ MXene for wastewater treatment and electrochemical energy storage. Sustain. Energy Fuels.

[115] Shpigel N., Malchik F., Levi M.D., Gavriel B., Bergman G., Tirosh S., Leifer N., Goobes G., Cohen R., Weitman M. (2020). New aqueous energy storage devices comprising graphite cathodes, MXene anodes and concentrated sulfuric acid solutions. Energy Storage Mater..

[116] Said Z., Sharma P., Aslfattahi N., Ghodbane M. (2022). Experimental analysis of novel ionic liquid-MXene hybrid nanofluid’s energy storage properties: Model-prediction using modern ensemble machine learning methods. J. Energy Storage.

[117] Zahra S.A., Murshed M.M., Naeem U., Gesing T.M., Rizwan S. (2023). Cation-assisted self-assembled pillared V_2_CT_x_ MXene electrodes for efficient energy storage. Chem. Eng. J..

[118] Aslfattahi N., Saidur R., Arifutzzaman A., Sadri R., Bimbo N., Sabri M.F.M., Maughan P.A., Bouscarrat L., Dawson R.J., Said S.M. (2020). Experimental investigation of energy storage properties and thermal conductivity of a novel organic phase change material/MXene as A new class of nanocomposites. J. Energy Storage.

[119] Gowthami D., Sharma R., Khalid M. (2023). 2D MXene based nanocomposites for solar driven renewable energy storage utilizing binary eutectic phase change material. J. Mol. Liq..

[120] Wang H.-Q., Wang J.-W., Wang X.-Z., Gao X.-H., Zhuang G.-C., Yang J.-B., Ren H. (2022). Dielectric properties and energy storage performance of PVDF-based composites with MoS_2_@MXene nanofiller. Chem. Eng. J..

[121] Shafique R., Rani M., Batool K., Shah A.A., Bahajjaj A.A.A., Sillanpää M., Alsalmah H.A., Janjua N.K., Arshad M. (2024). Nanoengineering of novel MXene (Ti_3_C2T_x_) based MgCr_2_O_4_ nanocomposite with detailed synthesis, morphology and characterization for enhanced energy storage application. Mater. Sci. Eng. B.

[122] Bukhari H., Iqbal A.M., Awan S.U., Hussain D., Shah S.A., Rizwan S. (2023). Intercalation of C60 into MXene Multilayers: A Promising Approach for Enhancing the Electrochemical Properties of Electrode Materials for High-Performance Energy Storage Applications. ACS Omega.

[123] Liu X., Lin F., Zhang X., Liu M., Sun Z., Zhang L., Min X., Mi R., Huang Z. (2021). Paraffin/Ti_3_C_2_T_x_ Mxene@ Gelatin aerogels composite Phase-Change materials with high Solar-Thermal conversion efficiency and enhanced thermal conductivity for thermal energy storage. Energy Fuels.

[124] Tian Y., Que W., Luo Y., Yang C., Yin X., Kong L.B. (2019). Surface nitrogen-modified 2D titanium carbide (MXene) with high energy density for aqueous supercapacitor applications. J. Mater. Chem. A.

[125] Cao Y., Li W., Huang D., Zhang J., Lin P., Zhang L., Sheng X., Chen Y., Lu X. (2022). One-step construction of novel phase change composites supported by a biomass/MXene gel network for efficient thermal energy storage. Sol. Energy Mater. Sol. Cells.

[126] Jones W.D. (2020). Carbon Capture and Conversion.

[127] Sabri M.A., Al Jitan S., Bahamon D., Vega L.F., Palmisano G. (2021). Current and future perspectives on catalytic-based integrated carbon capture and utilization. Sci. Total Environ..

[128] Morales-Salvador R., Gouveia J.D., Morales-Garcia A., Vines F., Gomes J.R., Illas F. (2021). Carbon capture and usage by MXenes. ACS Catal..

[129] Persson I., Halim J., Lind H., Hansen T.W., Wagner J.B., Näslund L.Å., Darakchieva V., Palisaitis J., Rosen J., Persson P.O. (2019). 2D transition metal carbides (MXenes) for carbon capture. Adv. Mater..

[130] Guo Z., Li Y., Sa B., Fang Y., Lin J., Huang Y., Tang C., Zhou J., Miao N., Sun Z. (2020). M2C-type MXenes: Promising catalysts for CO_2_ capture and reduction. Appl. Surf. Sci..

[131] Li N., Chen X., Ong W.-J., MacFarlane D.R., Zhao X., Cheetham A.K., Sun C. (2017). Understanding of electrochemical mechanisms for CO_2_ capture and conversion into hydrocarbon fuels in transition-metal carbides (MXenes). ACS Nano.

[132] Cui Y., Cao Z., Zhang Y., Chen H., Gu J., Du Z., Shi Y., Li B., Yang S. (2021). Single-Atom Sites on MXenes for Energy Conversion and Storage. Small Sci..

[133] Liu F.-Q., Liu X., Sun L., Li R., Yin C.-X., Wu B. (2021). MXene-supported stable adsorbents for superior CO_2_ capture. J. Mater. Chem. A.

[134] Bharath G., Rambabu K., Hai A., Othman I., Ponpandian N., Banat F., Show P.L. (2021). Hybrid Pd50-Ru50/MXene (Ti_3_C_2_T_x_) nanocatalyst for effective hydrogenation of CO_2_ to methanol toward climate change control. Chem. Eng. J..

[135] Hu Z., Xie Y., Yu D., Liu Q., Zhou L., Zhang K., Li P., Hu F., Li L., Chou S. (2021). Hierarchical Ti_3_C_2_T_x_ MXene/Carbon Nanotubes for Low Overpotential and Long-Life Li-CO_2_ Batteries. ACS Nano.

[136] Zhao Q., Zhang C., Hu R., Du Z., Gu J., Cui Y., Chen X., Xu W., Cheng Z., Li S. (2021). Selective etching quaternary MAX phase toward single atom copper immobilized MXene (Ti_3_C_2_Cl_x_) for efficient CO_2_ electroreduction to methanol. ACS Nano.

[137] Aliyu M., Yusuf B.O., Abdullah A., Bakare A.I., Mustapha U., Hakeem A.S., Ganiyu S.A. (2024). Ti_2_C-MXene/activated carbon nanocomposite for efficient CO_2_ capture: Insights into thermodynamics properties. Sep. Purif. Technol..

[138] Arifutzzaman A., Musa I.N., Aroua M.K., Saidur R. (2023). MXene based activated carbon novel nano-sandwich for efficient CO_2_ adsorption in fixed-bed column. J. CO_2_ Util..

[139] Luo W., Niu Z., Mu P., Li J. (2022). Pebax and CMC@MXene-Based Mixed Matrix Membrane with High Mechanical Strength for the Highly Efficient Capture of CO_2_. Macromolecules.

[140] Wu X., Fernandes D., Feron P., Ding M., Xu H., Xie Z. (2022). Production of cooling water by Ti_3_C_2_T_x_ MXene interlayered forward osmosis membranes for post-combustion CO_2_ capture system. J. Membr. Sci..

[141] Govindan B., Madhu R., Abu Haija M., Kusmartsev F.V., Banat F. (2022). Pd-Decorated 2D MXene (2D Ti_3_C_2_Ti_x_) as a High-Performance Electrocatalyst for Reduction of Carbon Dioxide into Fuels toward Climate Change Mitigation. Catalysts.

[142] Hu Z., Yang Y., Zhang X.-F., Xu C., Yao J. (2023). Integrating two-dimensional MXene fillers into nanocellulose for the fabrication of CO_2_ separation membranes. Sep. Purif. Technol..

[143] Wang D., Xin Y., Wang Y., Li X., Wu H., Zhang W., Yao D., Wang H., Zheng Y., He Z. (2021). A general way to transform Ti_3_C_2_T_x_ MXene into solvent-free fluids for filler phase applications. Chem. Eng. J..

[144] Shamsabadi A.A., Isfahani A.P., Salestan S.K., Rahimpour A., Ghalei B., Sivaniah E., Soroush M. (2019). Pushing rubbery polymer membranes to be economic for CO_2_ separation: Embedment with Ti_3_C_2_T_x_ MXene nanosheets. ACS Appl. Mater. Interfaces.

[145] Lin H., Gong K., Hykys P., Chen D., Ying W., Sofer Z., Yan Y., Li Z., Peng X. (2021). Nanoconfined deep eutectic solvent in laminated MXene for efficient CO_2_ separation. Chem. Eng. J..

[146] Luo W., Niu Z., Mu P., Li J. (2022). MXene/poly (ethylene glycol) mixed matrix membranes with excellent permeance for highly efficient separation of CO_2_/N_2_ and CO_2_/CH_4_. Colloids Surf. A.

